# Protective effect of silymarin on doxorubicin-induced cardiotoxicity in rats

**DOI:** 10.1186/2050-6511-13-S1-A9

**Published:** 2012-09-17

**Authors:** Nebojša Stilinović, Aleksandar Rašković, Momir Mikov, Saša Vukmirović, Boris Milijašević

**Affiliations:** 1 Department of Pharmacology, Toxicology and Clinical Pharmacology, Faculty of Medicine, University of Novi Sad, 21000 Novi Sad, Serbia

## Background

Silymarin, an extract of *Silybum marianum* seeds, possesses a broad spectrum of action: antifibrotic, antiinflammatory, lipid peroxidation-inhibiting and free radical-scavenging effects. It is a complex of five major compounds, and silibinin is the most biologically active component of the complex. The aim of this study was to investigate, evaluate and confirm the potential antioxidative effects of silymarin rich in silibinin.

## Methods

White laboratory rats of Wistar type were used in this experiment. They were treated with saline, 1 ml/kg, orally; olive oil 1 ml/kg, orally; silymarin, 60 mg/kg, orally, every day; with doxorubicin, 1.66 mg/kg, intraperitoneally, every second day; and with the combination of silymarin and doxorubicin in the stated doses. The animals were anaesthetised with urethane and a prepared jugular vein was connected to an infusion pump with verapamil in the course of recording an electrocardiogram. Then, animals were sacrificed by cardiopunction and blood samples were taken to determine serum enzymes activity.

## Results

The results show that the treatment with silymarin and doxorubicin in combination causes statistically significant increase of the verapamil dose necessary to produce first change (2.21:0.62, p < 0.01); continuous reaction (2.77:1.5, p < 0.05) and toxic effect (3.38:1.87, p < 0.05) in comparison to the groups treated with doxorubicin alone. The administration of silymarin prevented an increase of creatine kinase activity induced by doxorubicin (279:616; p < 0.01). The activity of lactate dehydrogenase was not significantlly different between groups. Silymarin also prevented the doxorubicin-induced increase in aspartate aminotransferase activity (349.5:279.5, p < 0.05).

## Concusions

On the basis of the results it can be concluded that pro-oxidative activity of doxorubicin is significantly lower when it is administered in combination with silymarin.

